# Knockdown of Glutamate Cysteine Ligase Catalytic Subunit by siRNA Causes the Gold Nanoparticles-Induced Cytotoxicity in Lung Cancer Cells

**DOI:** 10.1371/journal.pone.0118870

**Published:** 2015-03-19

**Authors:** Min Liu, Yunxue Zhao, Xiumei Zhang

**Affiliations:** Department of Pharmacology, School of Medicine, Shandong University, Jinan, Shandong, PR China; Universidade Nova de Lisboa, PORTUGAL

## Abstract

Gold nanoparticles (GNPs) have shown promising medical applications in cancer treatment involved in the regulation of intracellular redox balance. Previously, we have reported that GNPs can trigger apoptosis and necrosis in human lung cancer cells (A549) when L-buthionine-sulfoximine (BSO) was used to decrease the expression of intracellular glutathione (GSH). Herein, we investigated the cytotoxicity of GNPs toward lung cancer cells under the glutamate cysteine ligase catalytic subunit (GCLC) was silenced by siRNA. Our results showed that GNPs cause apoptosis and necrosis in cells transfected with GCLC siRNA by elevating intracellular reactive oxygen species (ROS). These findings demonstrated that the regulation of glutathione synthesis by GCLC siRNA in A549 cells can initiate the gold nanoparticles-induced cytotoxicity.

## Introduction

Recently, the interest in gold nanoparticles (GNPs) for cancer diagnosis and therapy, such as drug delivery carriers [1], cell targeting vectors [2], imaging [3], radiosensitization [4–7], and non-invasive ablation therapies [8, 9] has grown significantly. GNPs offer advantages in these applications because of their excellent biocompatibility [10], strong light absorption and scattering effect [11], high photothermal conversion rate and photostability [12–14], facile bioconjugation and biomodification [15]. Furthermore, the use of GNPs as anti-cancer agents has been extensively studied. Various attempts to incorporate GNPs into cancer treatments have been made.

Reduced glutathione (GSH), the most abundant intracellular thiol, is important in maintaining intra-cellular redox balance and is involved in the detoxification of exogenous and endogenous substances such as xenobiotics, ionizing radiation, organic peroxides and heavy metals [16,17]. It has been demonstrated that an aggressive tumor can be sensitive to drugs by using a therapy based on the modulation of GSH levels in cancer cells [18]. It is well known that glutathione is synthesized from its constituent amino acids in two sequential, catalysed by glutamylcysteine synthetase (GCL) and GSH synthase. GCL consists of a catalytic subunit (GCLC) and a modulatory subunit (GCLM), which catalyzes the first and rate-limiting step and plays a key role in glutathione homeostasis [19]. The intracellular GSH levels can be depleted through the specific inhibition of GCL. L-buthionine-sulfoximine (BSO), an inhibitor of GCL, is known to deplete the intracellular pool of glutathione and thereby cause oxidative stress [20]. Alterations in the specific activities of enzymes involved in GSH metabolism in the cancer cells have been implicated in oxidative stress and the depletion in GSH may increase the susceptibility of cancer cells to other harmful events [21–23]. We have reported previously that GNPs display cytotoxicity to lung cancer cells when L-buthionine-sulfoximine (BSO) was used to decrease the expression of intracellular glutathione [24]. So, gold nanoparticles may be employed as potential therapeutics by regulating the levels of glutathione in cancer cells. While, BSO is a kind of exogenous compounds. The influence of BSO on cells function is unpredictable. In the present work, we evaluated the effect of GCLC siRNA on GNPs-induced cytotoxity in lung cancer cells.

To our knowledge, there is no study on evaluating the roles of GCLC siRNA in GNPs-induced cell death. The primary objective of this study is to characterize the cytotoxity of GNPs in lung cancer cells when GCLC was knocked down by siRNA.

## Materials and Methods

### Cell culture

A549 cells (Shanghai Cell Bank, Type Culture Collection Committee, Chinese Academy of Sciences, cat number: TCHu150) were maintained in RPMI-1640 medium containing 4.5g/L glucose, 2mM L-glutamine, 1mM sodium pyruvate, 10% heat inactivated fetal bovine serum (FBS), 100U/mL penicillin and 100μg/mL streptomycin. Cells were grown in 5% CO_2_ at 37°C under a humidified atmosphere.

### Transfection of siRNA in A549 cell line

Gene silencing of GCLC was performed using a siRNA knockdown system. A non-specific control siRNA duplex [5′-UUCUCCGAACGUGUCACGUTT-3′], GCLC siRNA-1 duplex [5′-GCUAAUGAGUCUGACCAUU (dTdT)-3′], GCLC siRNA-2 duplex [5′-CUAUGUGGUGU UUGUGGUA (dTdT)-3′] and GCLC siRNA-3 duplex [5′-GUAGUAUUCUGAACUACCU (dTdT)-3′] were purchased from the Sigma—Aldrich. In brief, A549 cells were plated into 6-well plates at a density of 1.5×10^5^ per well. The next day, cells (~60–70% confluence) in each well were transfected with the negative control, GCLC siRNA (75pmol in free of FBS RPMI1640) using Thermo Scientific DharmaFECT Transfection Reagents (Thermo Scientific) according to the manufacturer’s instructions. One day later, the medium was changed to normal growth medium and the cells were cultured for an additional 48 hours. The transfected cells were collected and used for PCR, western blot analysis, and GSH levels measurement [25–27].

### RNA preparation and semiquantitative RT-PCR

Semiquantitative RT-PCR with β-actin as an internal control was performed to examine the alteration in the mRNA expression of GCLC in A549 cells transfected with siRNAs. Total RNA was extracted using Trizol reagent (Invitrogen) according to the manufacturer’s instructions [28]. The isolated RNA (2μg) from siRNAs treated cells was reverse transcribed into single-stranded cDNA in a reaction mixture containing: 10mmol/L dNTP, 1μg oligo (dT) primer, 20IU RNasin and 200IU M-MLV reverse transcriptase. PCR amplification was carried out with 0.4μg cDNA in a reaction volume containing 3pmol of each specific oligonucleotide primer, 10mmol/L dNTP, and 1.5IU Taq DNA polymerase. For all of the reactions, preliminary experiments were performed to determine the number of PCR cycles at which saturation occurred, and the experiments mentioned were carried out with a number of cycles that precede saturation. The sequences of the primers for GCLC and β-actin expression analyses were: GCLC, forward primer: 5′-TCCAGGTGACATTCCAAGCC-3′; reverse primer: 5′-GAAATCACTCCCCAGCGACA-3′. The thermal cycler unit was programmed for 36 cycles at 95°C for 30 seconds, 60°C for 30 seconds, and 72°C for 30 seconds. β-actin, forward primer: 5′-TGACGTGGACATCCGCAAAG-3′; reverse primer: 5′-CTGGAAGGTGGACAGCGAGG-3′. PCR products were electrophoresed on a 2% agarose gel (Sigma—Aldrich) and visualized after ethidium bromide staining over UV light [29].

### Western blotting

For GCLC detection, cells grown in six-well plates were incubated with negative control duplex and GCLC siRNA duplex for 24 hours, then cultured with normal growth medium for additional 48 hours. Subsequently, cells were washed with PBS, trypsinized and collected by centrifugation. A quantity of 30μg protein of each sample were mixed with loading buffer, incubated at 100°C for 5 minutes and separated on a 10% SDS—PAGE. Then, the samples were transferred onto a PVDF (polyvinylidene fluoride) membrane and blocked with 5% (w/v) skim milk dissolved in TBS + 0.05% (v/v) Tween-20 (TBST) for one hour at room temperature and probed with GCLC (1:500, Santa Cruz Biotechnology), or β-actin (1:2000, Santa Cruz Biotechnology) at 4°C overnight. Following, the membranes were incubated with a secondary antibody (1:4000 anti-rabbit, Cell Signaling Technology) for one hour, washed 3 times with TBST and the bands were visualized using ECL reagent (Thermo Scientific) [30].

### Total intracellular glutathione (GSH) levels

Cellular GSH content was analyzed using Glutathione Quantification Kit (Beyotime Institute Biotechnology). 5, 5’-dithiobis (2-nitrobenzoic acid) (DTNB) and GSH react to generate 2-nitro-5-thiobenzoic acid and glutathione disulfide (GSSG). Because 2-nitro-5-thiobenzoic acid is a yellow colored product, GSH concentration in samples can be measured at 412 nm absorbance with a multiwell plate reader. In brief, cells were transfected with negative control or three GCLC siRNA duplex for 24 hours, then cultured in normal growth medium for additional 48 hours. At the end of treatment, cells were harvested and then pelleted by centrifugation at 1000 rpm for 5 min, resuspended in lysis buffer containing 0.2% Triton X-100. Cell lysates were centrifuged at 13,000g for 10 min at 4°C, and the supernatants were used for the determination of total glutathione concentrations. GSH depletion in GCLC siRNA treated cells was indicated [31].

### Cytotoxicity measurement

Cytotoxicity was determined via cell counting. We observed that the inhibition of GCLC siRNA-1duplex is the most significant among three GCLC siRNA duplex. So, A549 cells were incubated with negative control or GCLC-specific siRNA-1, and then exposed to different doses of GNPs for additional 72 hours. Finally, cells were washed with PBS, trypsinized with trypsin/EDTA solution, and resuspended to a final volume of a 2 mL cell medium for further measurement of cell viability. The cell numbers in each sample were counted under a microscope using a Counting Chamber Set (Qiujing Inc) [32].

### Intracellular reactive oxygen species measurement

The production of ROS was measured using 2, 7-dichlorodihydro fluorescent diacetate (DCFH-DA, Beyotime Institute Biotechnology). DCFH-DA passively enters the cells, cellular esterases act on the molecule to form the non-fluorescent moiety DCFH, which is ionic in nature and, therefore, trapped inside the cells. A reaction with ROS leads to an oxidation of DCFH to the highly fluorescent compound dichloroflourescein (DCF). Briefly, cells grown in 6-well plates were transfected with the negative control, GCLC siRNA-1 using Thermo Scientific Dharma FECT Transfection Reagents. One day later, cells were treated with GNPs (20μM), GNPs (20μM) and exogenous GSH (1mM) or GNPs (20μM) and ROS scavenger N-acetyl-L-cysteine (NAC, 1mM) for additional 72 h. Then the cells were washed with PBS for three times and subsequently treated with 10μM DCFH-DA. After incubated for 30minutes, the cells were trypsinized, collected by centrifugation and resuspended in PBS. The fluorescence intensity of cells (50 000) from each well was measured with a fluorescence spectrophotometer (Thermo Scientific), with excitation and emission wavelengths of 488 and 525 nm, respectively [33].

### Flow cytometric analysis of apoptosis and necrosis

Cell counting result showed that GNPs had inhibitory function on the transfected cell survival. We investigated whether the reason of this inhibition is either early apoptosis or late apoptosis/necrosis. These effects were determined with fluorescein isothiocyanate (FITC)-labeled AnnexinV and PI staining (Invitrogen) by flow cytometric analysis. The transfected cells were treated with GNPs, GNPs and GSH, GNPs and NAC. After 72 h, cells were collected and washed twice with PBS, incubated with AnnexinV-FITC and PI for 15 minutes at room temperature in the dark. Apoptotic and necrotic cells were evaluated by flow cytometry. At least 1×10^4^ cells were analyzed per sample, and quadrant settings were based on the control samples. Living cells are negative for both propidium iodide (PI) and Annexin V, early apoptotic cells are PI negative, but AnnexinV positive, whereas late apoptotic/necrotic cells are positive for both PI and AnnexinV. All flow cytometry analyses were performed using commercially-available Cell Quest software (BD Bioscience).

### Mitochondrial membrane potential and intracellular cleaved caspase-3 assay

JC-1(5, 50, 6, 60-tetrachloro-1, 10, 3, 30-tetraethylbenzamidazolocarbocyanin iodide, Beyotime Institute Biotechnology) was used to evaluate the change in mitochondrial membrane potential as described previously [24]. A549 cells in the logarithmic growth phase were plated in 6-well cell culture plates and incubated for 24 hours, and then transfected with negative control or GCLC-specific siRNA-1. The transfected cells were treated with or without GNPs (20μM) for 72 hours, subsequently the cells were harvested and incubated with JC-1 for 30 minutes in darkness at 37°C. After staining, cells were washed twice with PBS and analysed by flow cytometry [34].

For intracellular cleaved caspase-3, the transfected cells that treated with or without GNPs were collected and fixed with 4% paraformaldehyde. After treated with 0.1% Triton X-100 and blocked with 1% BSA, cells were incubated with cleaved caspase-3 (Asp175) antibody (Alexa fluor 488 conjugate, Cell Signaling Technology) for 30 minutes. Then, the stained cells were analysed by flow cytometry [24].

### Statistical analysis

Statistical differences were evaluated using analysis of variance (ANOVA) and Student’s T-test with the Prism software (GraphPad Software, Inc.). P < 0.05 was considered statistically significant, and the data were labeled with (*) for p < 0.05, (**) for p < 0.01, and (***) for p < 0.001, respectively. Each group was tested in triplicate, and all experiments were performed at least three times.

## Results

### Efficacy of siRNAs against the catalytic GCL subunits

**Fig 1:**
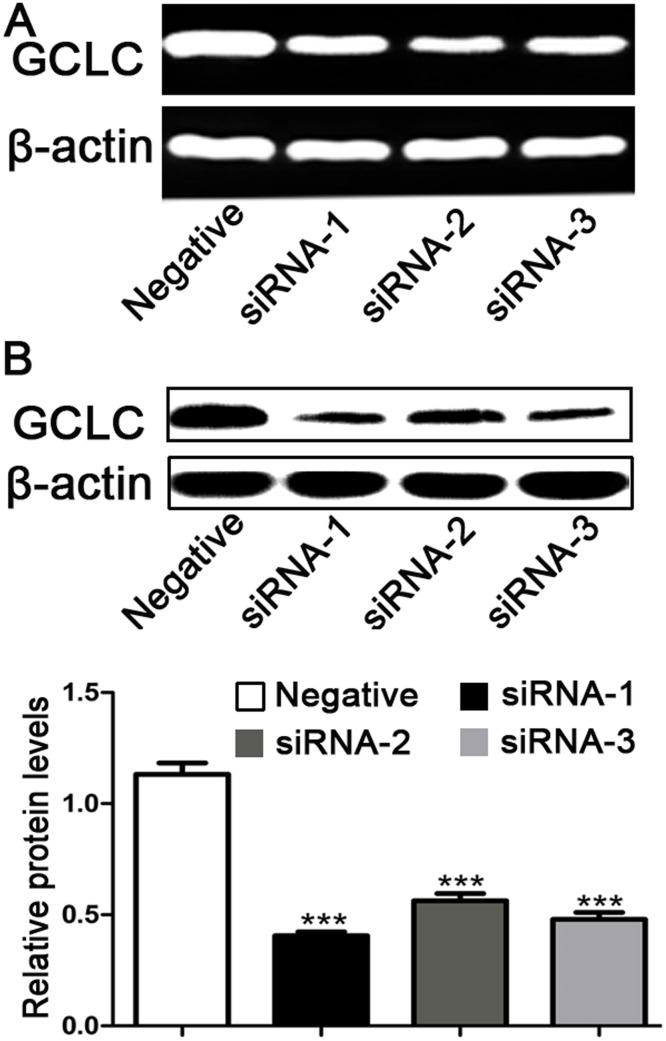
Knockdown of glutamate cysteine ligase catalytic subunit by siRNA. (A) GCLC mRNA levels in A549 cells following 24 hours transfected with negative control siRNA, GCLC siRNA-1, GCLC siRNA-2 and GCLC siRNA-3. GCLC siRNA significantly decreased the GCLC mRNA levels in A549 cells. (B) Representative Western blot gel documents for GCLC and summarized data showing that efficiency of gene silencing of GCLC by siRNA. Cytosolic proteins were isolated from transfected cells. GCLC protein levels in cell extracts were measured by Western blot analysis and were normalised to β-actin expression levels. *** P<0.001, compared to negative control.

To investigate the knockdown effect of three 19-nucleotide sequences for catalytic GCL subunits, A549 cells were transfected with negative control or GCLC siRNA for 24 hours, followed by cultured for an additional 48 hours in normal growth medium. Semiquantitative RT-PCR analysis was performed to examine the GCLC mRNA expression. The mRNA levels of GCLC were significantly reduced in A549 cells transfected with GCLC siRNA. In contrast, negative control-siRNA was less potent (Fig. 1A). In all cells transfected with GCLC siRNA, mRNA levels of GCLC were homogeneous without a significant deviation. Meanwhile, we estimated the efficacy of the GCLC siRNA by assaying their ability to interfere with the expressed proteins by Western blot. As shown in Fig. 1B, GCLC siRNA significantly inhibited the expression of GCLC protein in A549 cells. The three different siRNA for catalytic GCL subunits differentially disrupted the expression of the GCLC compared with negative control siRNA and the GCLC siRNA-1 was among the most effective.

### Effect of GCLC siRNA on intracellular GSH levels in A549 cells

**Fig 2:**
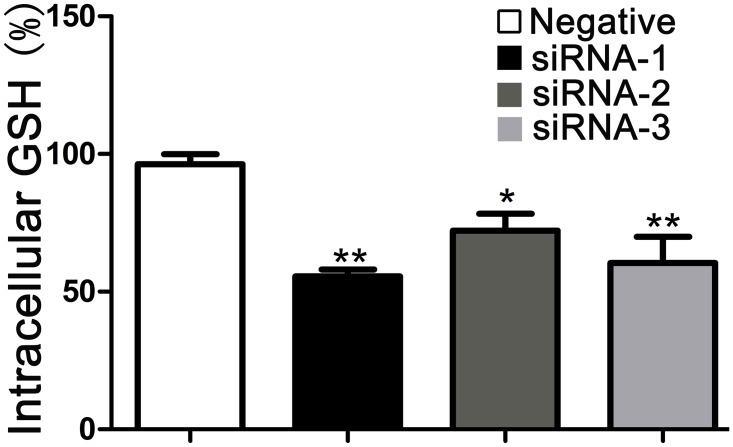
Effect of GCLC siRNA on intracellular GSH levels. Cytosol was isolated from cells transfected with negative control siRNA, GCLC siRNA-1, GCLC siRNA-2 and GCLC siRNA-3. The intracellular GSH levels were determined at 412 nm absorbance with a multiwell plate reader. Data represent the mean percentage of negative control (n = 3) ±SD for 4 independent experiments. *p<0.05, ** P<0.01, compared to negative control.

GCLC is essential for glutathione biosynthesis, which maintains glutathione concentrations in intact cells. We have shown that the siRNA targeted against catalytic GCL subunits decrease the mRNA levels of GCLC and inhibit expression of GCLC protein in A549 cells. To corroborate further the specificity and efficacy of GCLC siRNA, the effect of GCLC siRNA on intracellular GSH levels was measured [32]. As expected, compared with negative control group, GCLC siRNA obviously decreased the GSH levels and the GCLC siRNA-1 is the most significant (Fig. 2), which is correspondence with the results that we have observed above. In view of these results, GCLC siRNA-1 was used in the next experiments. Knock-down experiments demonstrated that GCLC siRNA influence the expression of GCLC and led to a significant reduction of GSH production.

### The GCLC siRNA regulates cell death induced by GNPs

**Fig 3:**
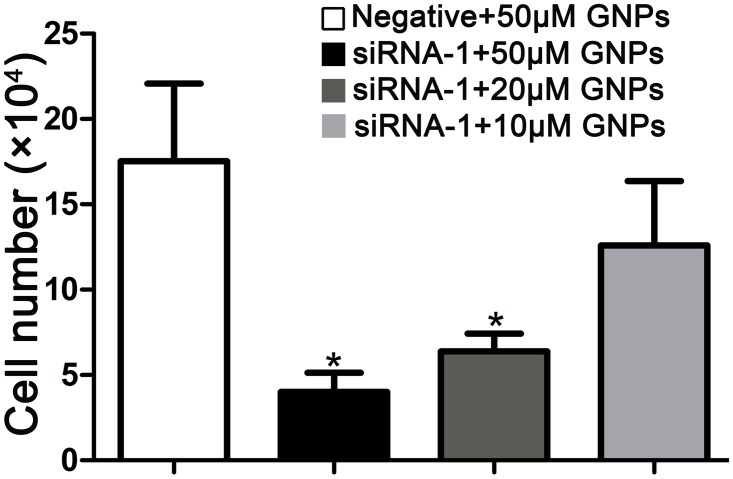
The cell growth is inhibited by GNPs in cells transfected with GCLC siRNA. The A549 cells were transfected with negative control or GCLC siRNA-1 for 24 hours, followed by cultured for an additional 3 days in the absence of GNPs, subsequently harvested and counted. Each bar represents the mean (±SD n = 4) of triplicate determinations. *p<0.05 versus negative control.

Total GSH content was decreased in a significant manner in A549 cells transfected with GCLC siRNA. When BSO was used to decrease the expression of glutathione in A549 cells, GNPs showed evident cytotoxicity to cells [32]. However, the effect of GCLC siRNA on the cytotoxicity of GNPs is still unknown. Next, we investigated whether GNPs altered A549 cells survival upon siRNA-mediated disruption of GCLC activity. Based on the results of knock-down experiments, A549 cells were transfected with negative control-siRNA or GCLC siRNA-1, and then exposed to various concentrations of GNPs. As control experiment, GCLC siRNA did not induce remarkable cytotoxicity on A549 cells (S1 Fig.). While, we observed that GNPs dose-dependently inhibited the growth of A549 cells pretreated with GCLC siRNA-1. Compared with negative control group, treatment of A549 cells with 50μM GNPs resulted in significantly decreased the population of viable cells after depleting intracellular GSH (Fig. 3).

### GNPs-induced cytotoxicity is associated with elevation of intracellular ROS in GCLC siRNA treated cells

**Fig 4:**
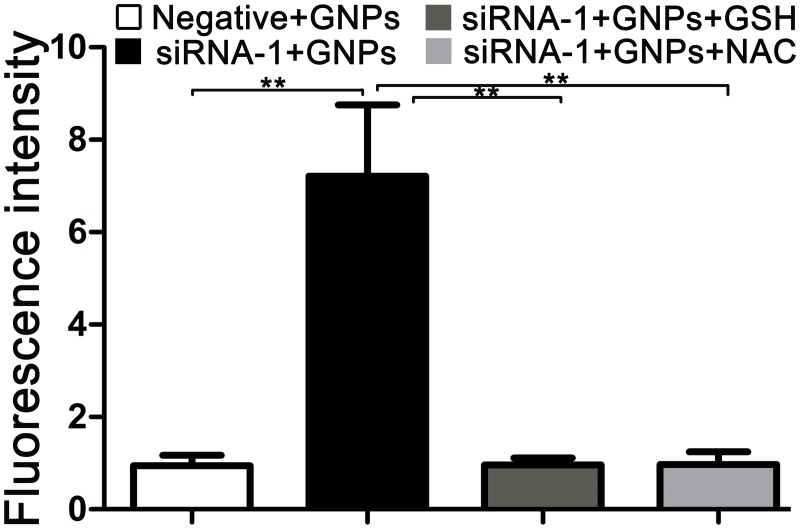
Effect of GNPs on intracellular ROS levels in GCLC siRNA pretreated cells. Exponentially growing cells were transfected with negative control and GCLC siRNA-1 for 24 hours, following treated with GNPs (20μM), GNPs (20μM) and GSH (1mM), GNPs (20μM) and NAC (1mM). ROS levels were measured. Graphs indicate ROS (as determined by DCF) levels (%) compared with negative control cells. Each bar represents the mean (±SD n = 4) of triplicate determinations. **p<0.01

Since ROS has been suggested as a critical factor in the determination of cell death mode. We further investigated whether ROS participate in the regulation of GNP-induced cell death when the cells were transfected with GCLC siRNA. As shown in Fig. 4, after silencing GCLC expression with siRNA-1, GNPs resulted in an approximately 7-fold increase of ROS levels compared with that in negative control group. However, when the cells were treated with GCLC siRNA alone, the influence of ROS production was not observed in the absence of GNPs (S2 Fig.). Exogenous GSH and NAC significantly suppressed GNPs-induced ROS production, which could protect GCLC siRNA treated cells from cytotoxicity of GNPs. These results mean that cytotoxicity of GNPs is at least partially associated with the increase of the ROS levels in GCLC siRNA-1 pretreated cells (Fig. 4).

### GNPs initiate apoptosis and necrosis in cells transfected with GCLC siRNA

**Fig 5:**
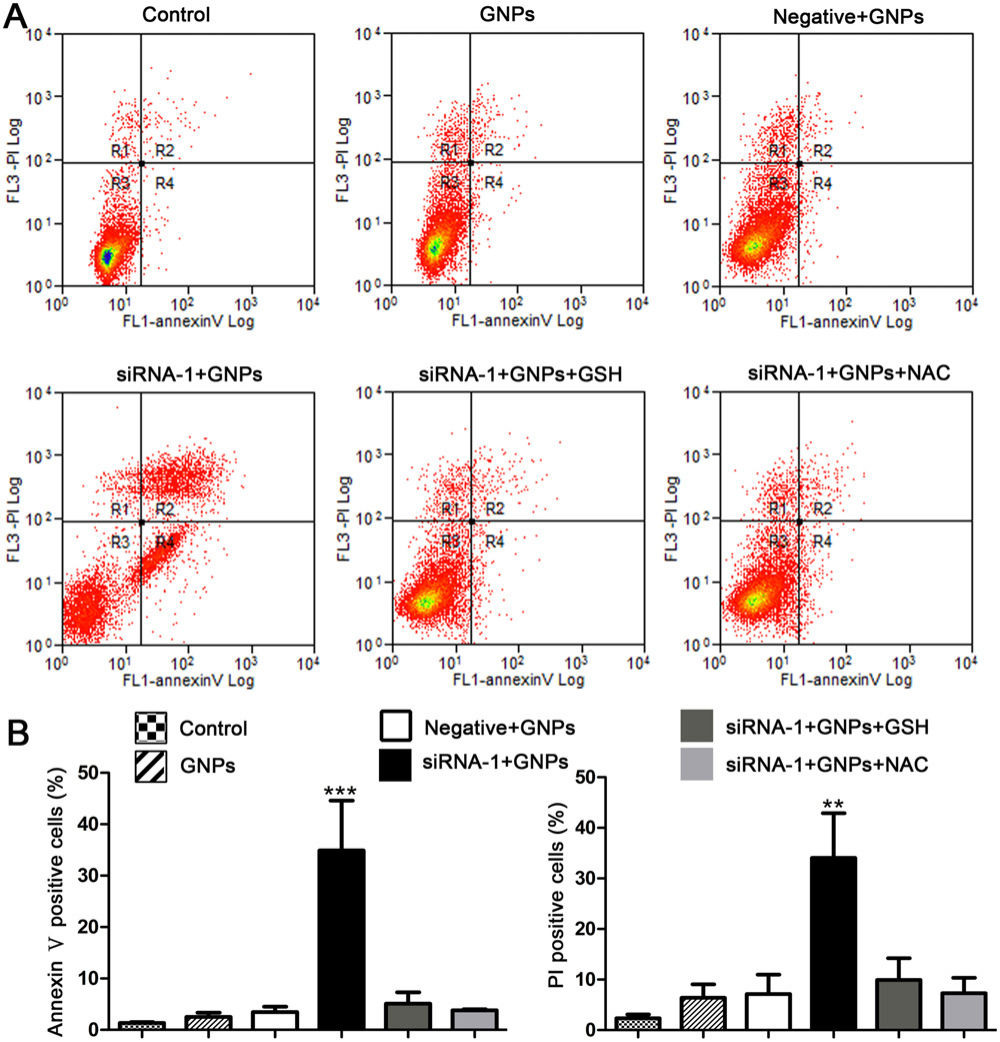
GNPs induce apoptosis and necrosis in cells transfected with GCLC siRNA. Cells were transfected with either non-target control siRNA or GCLC-specific siRNA-1. One day later, cells were treated with GNPs (20μM), GNPs (20μM)﹢GSH (1mM) and GNPs (20μM)+NAC(1mM) for additional 72 hours. AnnexinV-FITC and PI cells were measured with flow cytometry. (A) The fluorescence pattern of AnnexinV-FITC and PI-stained A549 cells after treatment. (B) Percentages of Annexin V positive or PI positive cells for different treatments. Each bar represents the mean (±SD n = 3). **p<0.01, ***P<0.001, versus control.

The evidence that GNPs induce cell death and enhance the production of intracellular ROS prompted us to further examine whether GNPs cause apoptosis and necrosis in GCLC siRNA treated cells. Dual staining of cells with Annexin V-FITC and PI was used to distinguish apoptotic and necrotic cells from normal cells. As shown in Fig. 5, GNPs increased the proportion of AnnexinV-stained cells when the GSH levels were decreased by silencing GCLC expression. The population of total PI positive cells, which comprises apoptosis and necrosis, was also significantly increased in GCLC siRNA and GNPs treated cells. Treatment with GCLC siRNA alone did not increased the population of apoptosis and necrosis as compared with control group (S3 Fig.). These data showed that GNPs relatively affect Annexin V-PI stained cell number in cells transfected with GCLC siRNA. To test if apoptosis or necrosis was activated by ROS, we employed the exogenous GSH and NAC. Treatment with GSH and NAC both significantly suppressed the appearance of Annexin V and PI positive cells induced by GNPs in GCLC siRNA treated cells (Fig. 5). These results indicated that GNPs initiate apoptosis and necrosis by elevating intracellular ROS levels in lung cancer cells pretreated with GCLC siRNA.

### GNPs cause the depolarization of mitochondrial membrane potential in GCLC siRNA treated cells

**Fig 6:**
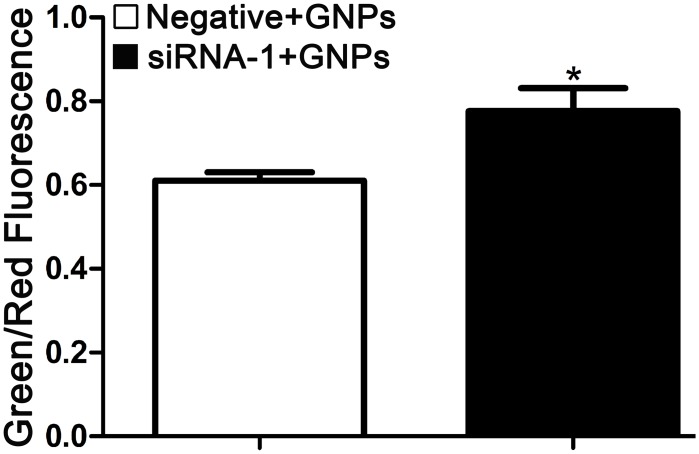
Flow cytometry analysis of mitochondrial membrane potential in GNPs-treated cells. After transfected with negative control siRNA or GCLC siRNA-1, cells were treated with GNPs (20μM) for additional 72 hours then collected and stained with JC-1 in darkness at 37°C, rinsed by PBS. The fluorescence shift (red to green) of samples was measured by flow cytometry. Each bar represents the mean (±SD n = 4) of triplicate determinations. *p<0.05, compared with negative control group.

Because depolarization of mitochondrial membrane potential (ΔΨm) plays a pivotal role in apoptosis [35], we examined whether GNPs altered mitochondrial membrane potential in cells transfected with GCLC siRNA-1. The fluorescent probe JC-1 dye was used to evaluate the change of mitochondrial membrane potential and the fluorescence shift (red to green) of samples was measured by flow cytometry. We observed that GNPs caused an increase in green/red fluorescence intensity in GCLC siRNA treated cells (Fig. 6). While, cells transfected with GCLC siRNA in the absence of GNPs showed no change of mitochondrial membrane potential as compare with control group (S4 Fig.). These results indicated that the induction of apoptosis and necrosis by GNPs in GCLC siRNA pretreated cells is closely associated with mitochondrial membrane potential.

### GNPs induce activation of caspase-3 in cells transfected with GCLC siRNA

**Fig 7:**
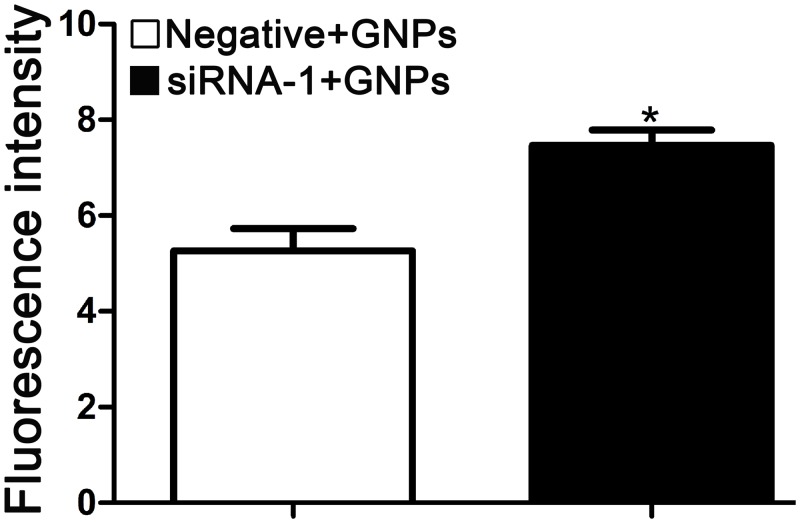
GNPs induce caspase activation in cells transfected with GCLC siRNA. Activation of caspase-3 was measured using specific antibodies by flow cytometry. Intracellular GSH was depleted by GCLC siRNA-1, after approximately 72 hours of GNPs treatment, the cells were collected, treated with 0.1% Triton X-100 and blocked with 1% BSA, then incubated with cleaved caspase-3 (Asp175) antibody (Alexa fluor 488 conjugate) for 30 minutes. The fluorescence intensity was measured by flow cytometry. Each bar represents the mean (±SD n = 4) of triplicate determinations. *p<0.05, compared with negative control group.

Mitochondrial-dependent apoptosis is initiated by recruitment and activation of caspase [36]. Thus, we further ask whether caspase-3 is activated during GNPs induced apoptosis in GCLC siRNA treated cells. When the labile cytosolic GSH pool was depleted by GCLC siRNA, cells were cultured with or without GNPs. The activation of caspase-3 was measured using specific antibodies that recognize the particularly cleaved and activated form by flow cytometry. As shown in Fig. 7, GNPs significantly increased caspase-3 activities in GCLC siRNA treated cells. GCLC siRNA alone hardly affected the activity of cascase-3 in comparison with control group (S5 Fig.). Therefore, GNPs induced apoptosis mainly through the mitochondrial-dependent caspase pathway.

## Discussion

Over the recent years, apoptosis induction due to alterations in intracellular redox status by certain oxidative or non-oxidative stimuli has become a focus of extensive research [23, 25, 37, 38]. ROS and GSH are the two major determinants of cellular redox equilibrium. ROS often cause cellular damage and lead to cell death, especially at high concentration. Pharmacological GSH depletion by BSO highly sensitizes tumor cells to apoptosis induced by chemotherapeutic agents. Hence, manipulation of cellular redox status represents a viable strategy for apoptosis induction by anti-cancer agents. Meanwhile, Gold nanoparticles in cancer diagnosis and treatment have become the international research hot spot. Previously, we found that GSH metabolic pathway is directly involved in the detoxification of GNPs [24]. When the intracellular GSH expression was inhibited by BSO, the cytotoxicity of GNPs was obvious. GNPs induce cell death by reactive oxygen species (ROS) generation in A549 cells with low intracellular glutathione [24, 32]. Geng F and colleagues have reported that the interaction of x-ray radiation with GNPs induced elevated levels of ROS production is one of the mechanisms by which GNPs can enhance radiotherapy on ovarian cancer [39]. Oxidative stress contributes to gold nanoparticle-induced cytotoxicity in human leukemia (HL-60) and hepatoma (HepG2) cells [40]. These results provided clear evidence that GNPs may be employed not only as carriers themselves but also as potential therapeutics by exploiting their capability to decrease intracellular GSH expression and generate cytotoxic responses. In this study, we adopt GCLC-specific siRNA to inhibit the expression of GCLC and lead to a significant decrease in GSH production, and then detected the cytotoxicity of GNPs. After treatment with GCLC siRNA, ROS was detected in the presence of GNPs in A549 cells. The result showed that GNPs can affect the ROS levels in GCLC siRNA treated cells. GSH and NAC can eliminate the ROS to neutralize the cytotoxicity of GNPs in lung cancer cells transfected with GCLC siRNA. These results indicted that after knocking down of GCLC by siRNA, ROS attributed to the cell death induced by GNPs in lung cancer cells.

It is known that divergent cell death pathways coexist in mammalian cells and their activation depends upon the type and intensity of the stimulus. A lot of importance has been given to the apoptotic and necrotic cell death in anti-tumor therapy [41–44]. Multiple modes of cell death are concurrently induced in GNPs-exposed lung cancer cells with low intracellular GSH, including apoptosis and necrosis. We observed that GNPs can induce apoptosis and necrosis simultaneously when the GCLC was silenced by siRNA in lung cancer cells. These findings suggested that both apoptosis and necrosis are important mechanism of GNPs-induced cell death.

To conclude, gold nanoparticles play more and more important role in the application of anti-tumor research. The gold nanoparticles once conjugated with GCLC-specific siRNA may effectively induce tumor cell death. However, more extensive and animal studies with different animal species are needed to take findings of this investigation to clinical trials.

## Supporting Information

S1 Fig Effect of GCLC siRNA on cell survival in A549 cells. Cells were seeded before transfected with GCLC siRNA, and then cultured in normal growth medium. The cell numbers were counted at 48-h intervals for cells viability. Each bar represents the mean (±SD n = 3) of triplicate determinations.
(TIF)

S2 Fig ROS production in A549 cells transfected with GCLC siRNA. Cells grown in 6-well plates were transfected with the negative control or GCLC siRNA. One day later, cells were cultured in normal growth medium for additional 48 h and subsequently treated with 10μM DCFH-DA. The fluorescence intensity of cells was measured with a fluorescence spectrophotometer. Bars represent mean (±SD n = 3) of triplicate determinations.
(TIF)

S3 Fig Apoptotic and necrotic response of A549 cells transfected with GCLC siRNA. After knockdown of GCLC expression, cells were stained with Annexin V-FITC and propidium iodide (PI) and analyzed by flow cytometry. Representative flow cytometric results are shown as dot plot.
(TIF)

S4 Fig Effect of GCLC siRNA on mitochondrial membrane potential (ΔΨm) in A549 cells. The cells treated with negative control or GCLC siRNA were stained with JC-1 for 20 min at 37°C. The fluorescence shift (red to green) of samples was then detected using flow cytometry. Data represent the mean (±SD n = 3) of three independent experiments.
(TIF)

S5 Fig Effect of GCLC knockdown on the activity of caspase-3 in A549 cells. Twenty-four hours after transfection with negative control or GCLC siRNA, cells were maintained in normal growth medium for additional 48h. Caspase-3 activities in samples were determined using cleaved caspase-3 (Asp175) antibody (Alexa fluor 488 conjugate) by flow cytometry. The values are expressed as the mean ± SD of three independent experiments.
(TIF)
