# Supplementary figures and images for: Knockdown of Glutamate Cysteine Ligase Catalytic Subunit by siRNA Causes the Gold Nanoparticles-Induced Cytotoxicity in Lung Cancer Cells
Source: PLoS One. 2015 Mar 19;10(3):e0118870. doi: 10.1371/journal.pone.0118870 (PMC4366198; doi:10.1371/journal.pone.0118870)

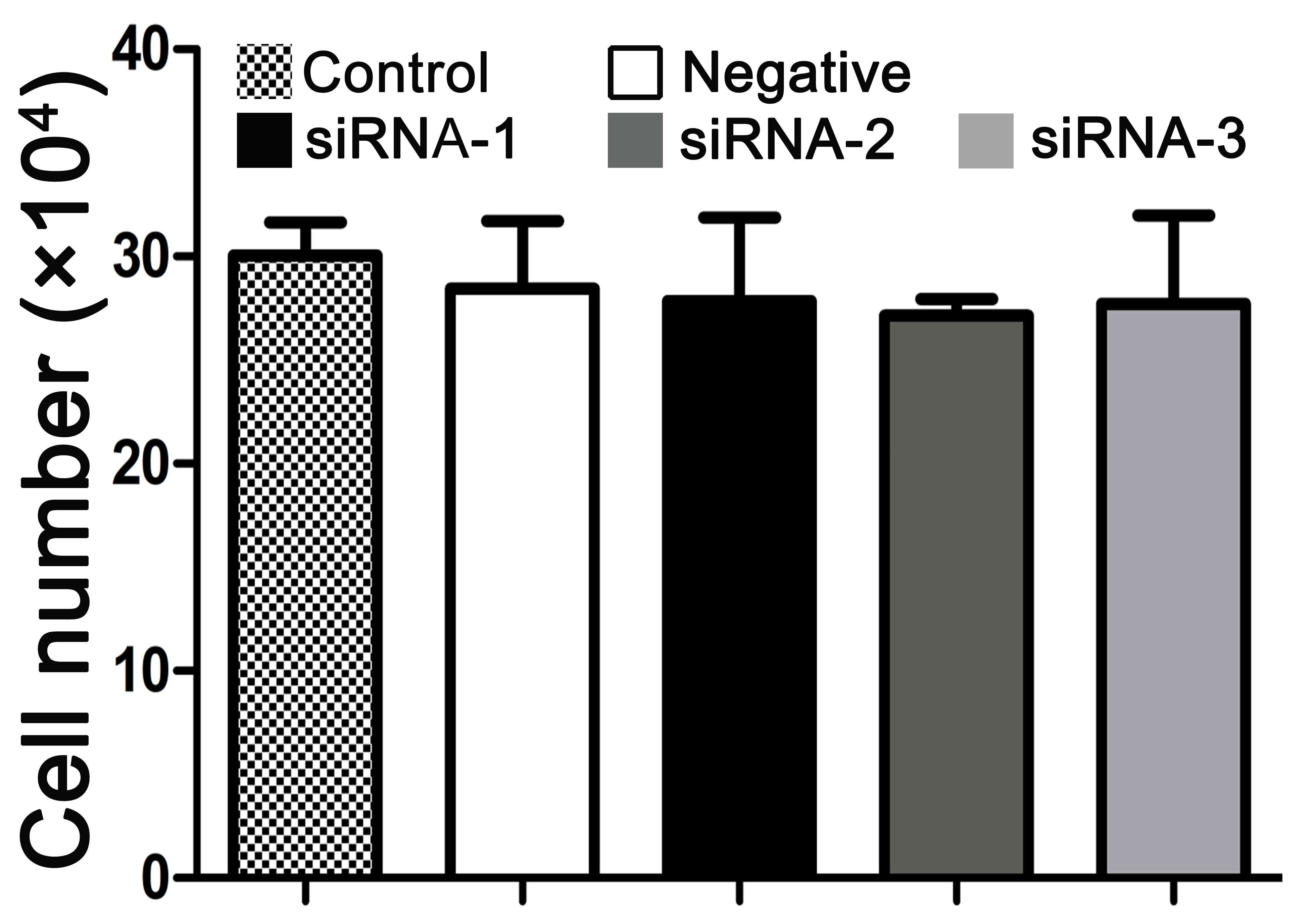

Supplement: S1 Fig — Cells were seeded before transfected with GCLC siRNA, and then cultured in normal growth medium. The cell numbers were counted at 48-h intervals for cells viability. Each bar represents the mean (±SD n = 3) of triplicate determinations. (TIF) [file pone.0118870.s001.tif]

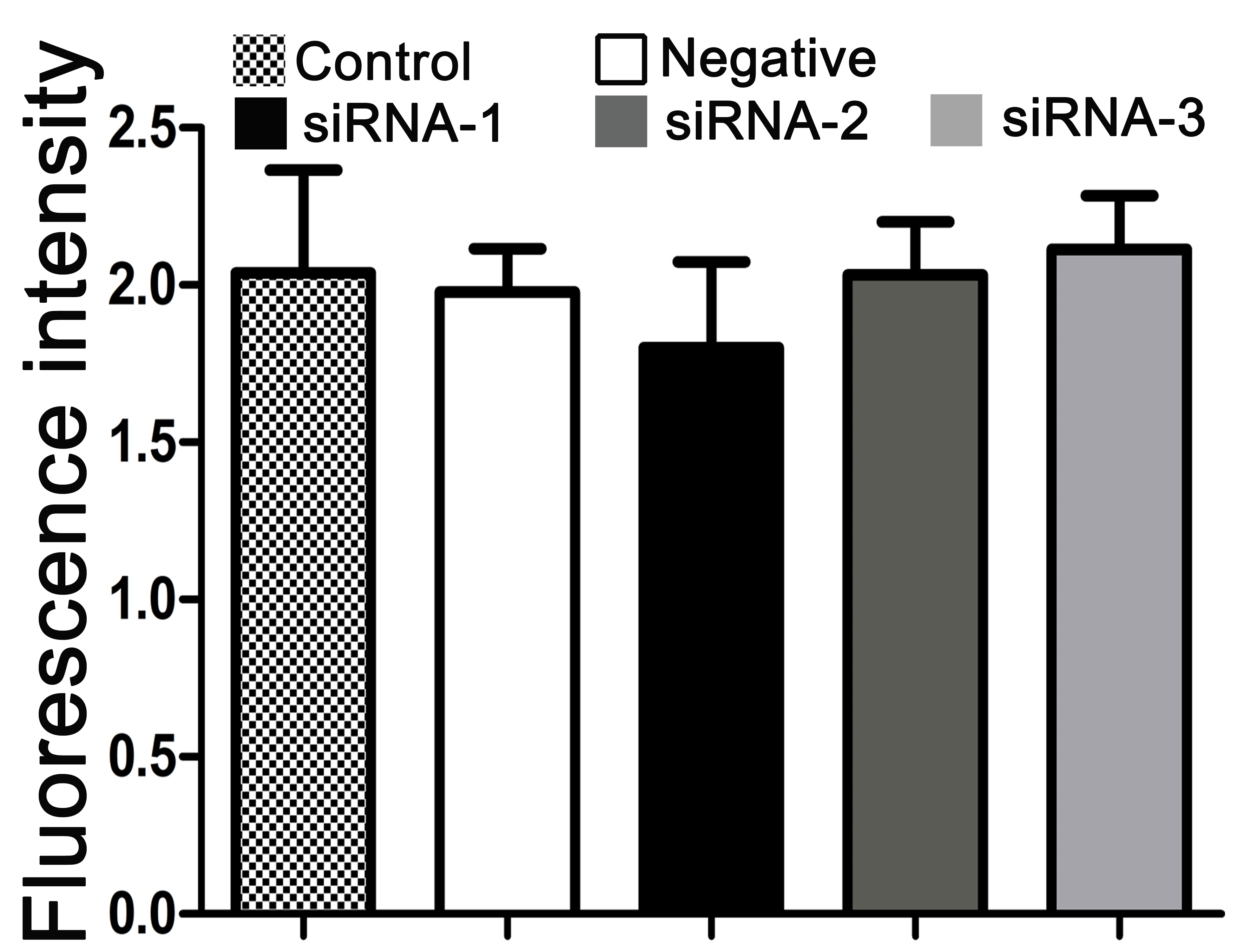

Supplement: S2 Fig — Cells grown in 6-well plates were transfected with the negative control or GCLC siRNA. One day later, cells were cultured in normal growth medium for additional 48 h and subsequently treated with 10μM DCFH-DA. The fluorescence intensity of cells was measured with a fluorescence spectrophotometer. Bars represent mean (±SD n = 3) of triplicate determinations. (TIF) [file pone.0118870.s002.tif]

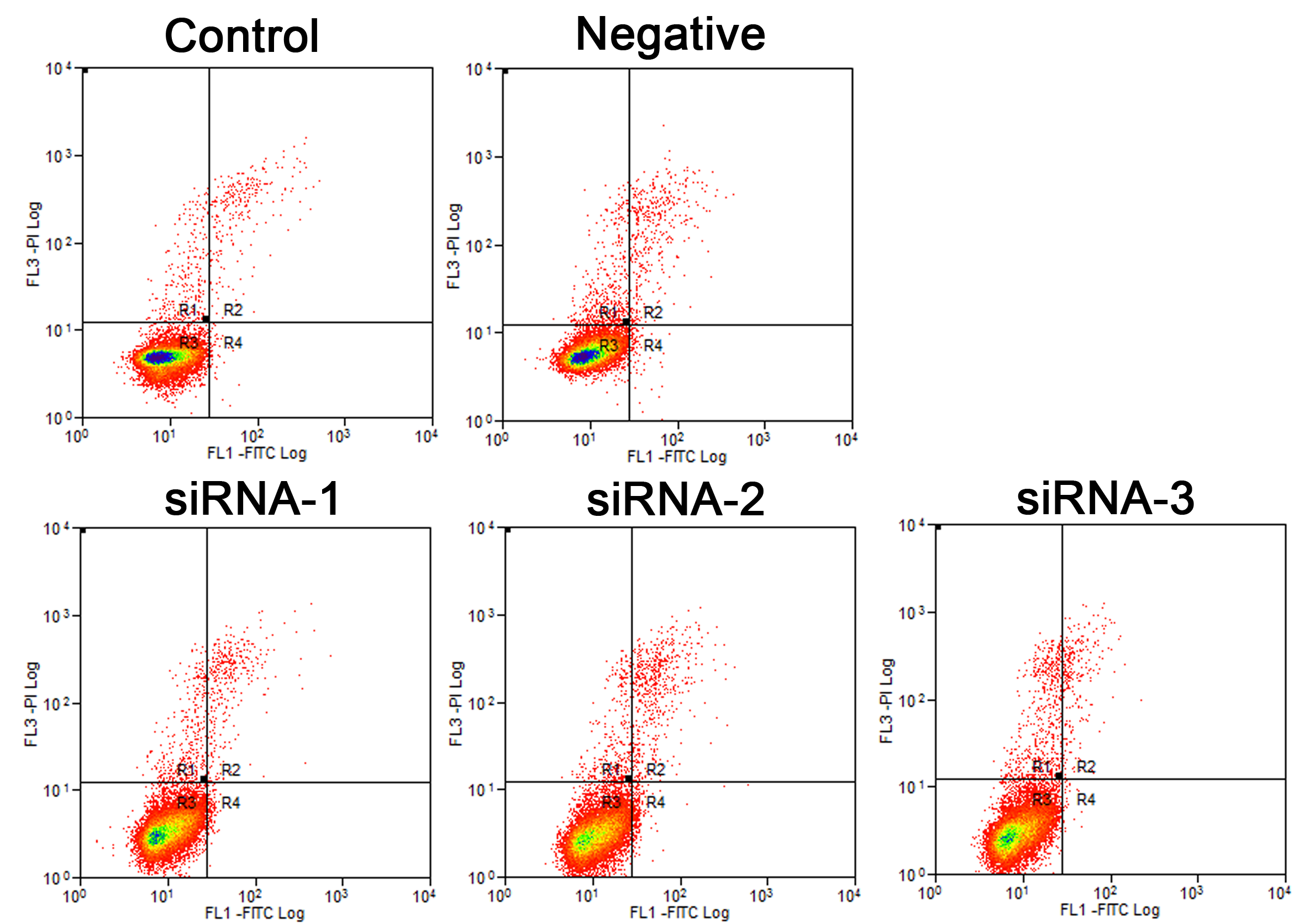

Supplement: S3 Fig — After knockdown of GCLC expression, cells were stained with Annexin V-FITC and propidium iodide (PI) and analyzed by flow cytometry. Representative flow cytometric results are shown as dot plot. (TIF) [file pone.0118870.s003.tif]

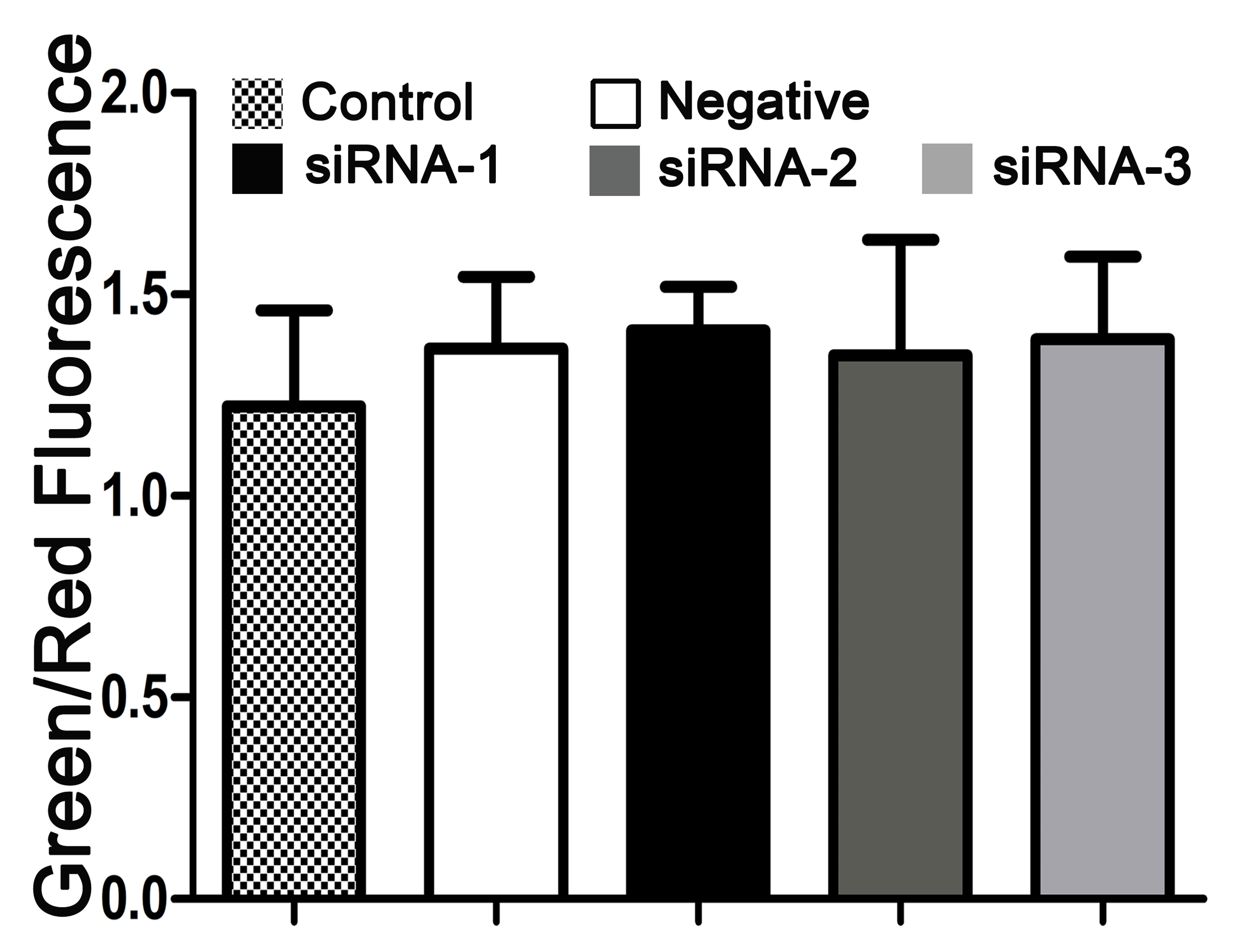

Supplement: S4 Fig — The cells treated with negative control or GCLC siRNA were stained with JC-1 for 20 min at 37°C. The fluorescence shift (red to green) of samples was then detected using flow cytometry. Data represent the mean (±SD n = 3) of three independent experiments. (TIF) [file pone.0118870.s004.tif]

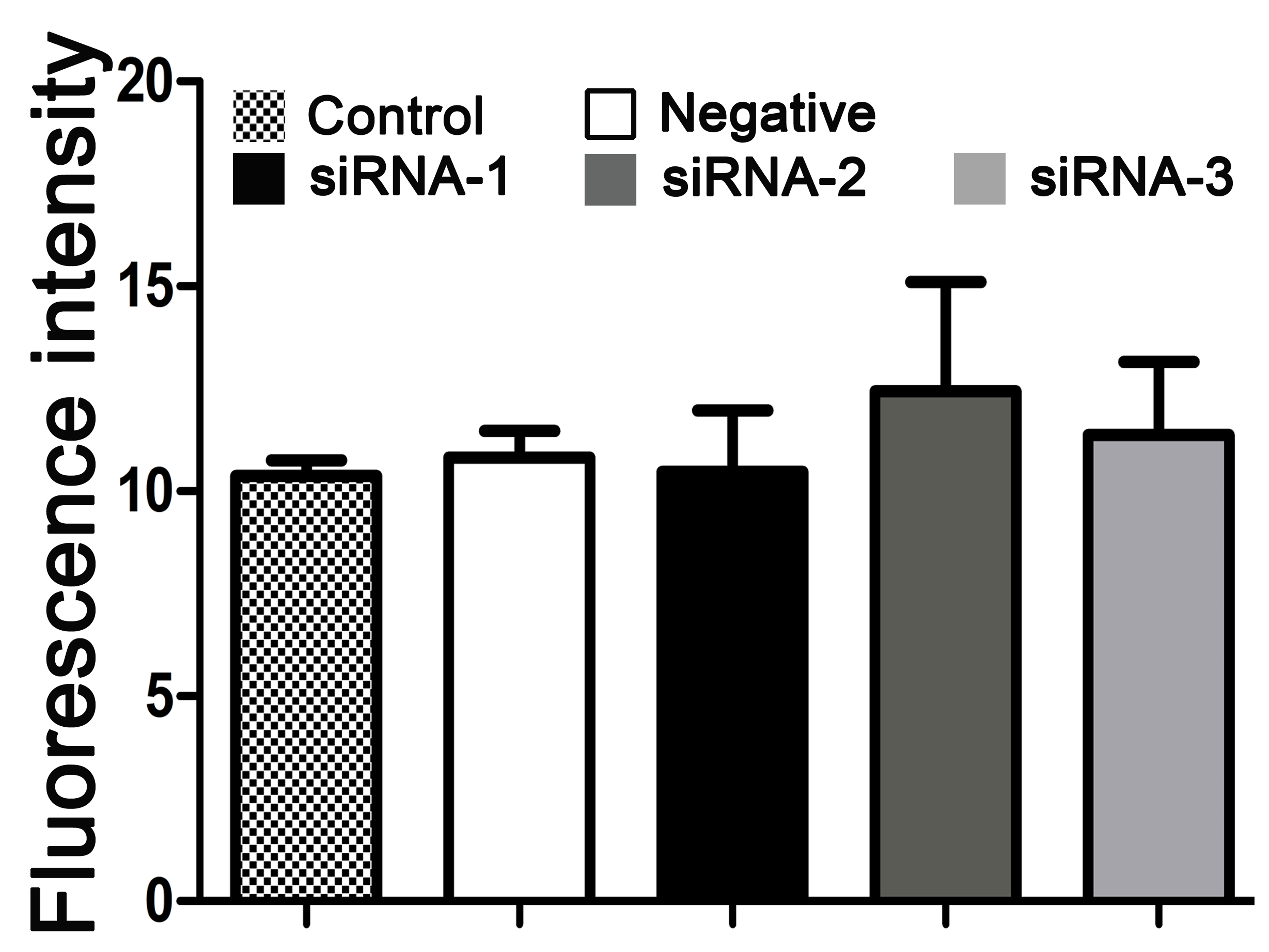

Supplement: S5 Fig — Twenty-four hours after transfection with negative control or GCLC siRNA, cells were maintained in normal growth medium for additional 48h. Caspase-3 activities in samples were determined using cleaved caspase-3 (Asp175) antibody (Alexa fluor 488 conjugate) by flow cytometry. The values are expressed as the mean ± SD of three independent experiments. (TIF) [file pone.0118870.s005.tif]
